# Selective prophylactic lateral node dissection improves the ipsilateral lateral node recurrence-free survival: A retrospective single-center cohort study

**DOI:** 10.1016/j.amsu.2020.07.046

**Published:** 2020-07-28

**Authors:** Makoto Fujishima, Akira Miyauchi, Yasuhiro Ito, Takumi Kudo, Minoru Kihara, Akihiro Miya

**Affiliations:** a Department of Surgery, Kuma Hospital, 8-2-35, Shimoyamate-dori, Chuo-ku, Kobe, 650-0011, Japan; b Department of Internal Medicine, Kuma Hospital, 8-2-35, Shimoyamate-dori, Chuo-ku, Kobe, 650-0011, Japan

**Keywords:** Prophylactic lateral neck dissection, Positive extrathyroidal extension, Papillary thyroid carcinoma, Recurrence, PTC, papillary thyroid carcinoma, pLND, Prophylactic lateral neck dissection, Ex, extrathyroidal extension, RFS, recurrence free survival, RAI, radioactive iodine

## Abstract

**Background:**

Some studies have shown that prophylactic lateral neck dissection (pLND) may be beneficial for patients with papillary thyroid carcinoma (PTC); however, none of the Western guidelines currently recommends this procedure. Since 2007, the decision to perform pLND at our institution has been made on a case-by-case basis with different risk factors in mind. In this study, we investigated the significance and indications of pLND in patients with PTC.

**Methods:**

We identified patients at stage N0 or N1a and M0 with PTC who underwent surgery from 2007 to 2017. We compared lateral compartment recurrence-free survival (RFS) and distant RFS between patients who did and did not undergo pLND (pLND and non-pLND groups).

**Results:**

pLND was performed in 494/3177 (15.5%) patients with PTC (tumor size [T] ≥2 cm, cN0 or N1a, M0). Overall, no significant difference in lateral compartment RFS was detected between the pLND and non-pLND groups. On multivariate analysis, T ≥ 3 cm and positive extrathyroidal extension were independent predictors for recurrence to the lateral compartment. In the subset analysis of T ≥ 3 cm with positive extrathyroidal extension (n = 127), the lateral compartment RFS rate of the pLND group was significantly better (p < 0.01) than that of the non-pLND group (p < 0.01). In this subset, pLND reduced recurrence to the lateral compartment by 20.7% during the 5-year follow-up. However, pLND did not improve distant RFS.

**Conclusions:**

pLND significantly improved lateral node RFS in patients having PTC ≥3 cm with significant extrathyroidal extension. For such patients, pLND at initial surgery may be considered to avoid second surgery.

## Highlights

• pLND may be beneficial for PTC.
• Western guidelines currently do not recommend this procedure.
• pLND improves ipsilateral lateral node RFS for patients with T ≥ 3 cm & positive Ex.
• pLND did not prevent distant recurrence.

## 1 Introduction

Previous studies demonstrated that if prophylactic lateral neck dissection (pLND) is performed for in those with lateral node-negative (cN0 or cN1a) papillary thyroid carcinoma (PTC), the incidence of positive pathological lateral node metastasis is high [1,2]. In Western guidelines, pLND is not actively recommended for PTC [[3], [4], [5], [6]]. Madenci et al. performed a systematic review regarding the difference in prognosis between patients with well-differentiated carcinoma who underwent pLND and those who did not; however, due to data limitations, they only concluded that routine pLND did not have a clear advantageous risk-to-benefit ratio [7].

Previously in Japan, pLND for N0 or N1a PTC was very actively performed with the intension to reduce PTC recurrence at the lateral lymph nodes and distant organs. Currently, however, according to the Clinical Practice Guidelines on the Management of Thyroid Tumors of the Japan Association of Endocrine Surgeons/Japanese Society of Thyroid Surgery (the present Japan Association of Endocrine Surgery), pLND is not recommended for low-risk (T1N0M0) PTC, and for intermediate- and high-risk PTCs. The indication of pLND should instead be determined based on other prognostic factors, patient backgrounds, and their willingness to go through with the procedure [8]. Two previous Japanese studies investigated the advantages of pLND for patients with N0 or N1a PTC and showed that large tumor size (T), male gender, old age, significant extrathyroid extension (Ex), large node metastasis, and distant metastasis were risk factors for lymph node recurrence for N0 or N1a PTC [2,9]. However, neither of these were comparative studies between patients who did and did not undergo pLND.

Until 2005, like many other Japanese institutions, our institution performed pLND for those with PTC almost routinely with the intention to reduce PTC recurrence at the lateral lymph nodes and distant organs. Since 2007, we changed our policy, because we found that routine pLND did not improve the outcome and that male sex, T ≥ 3 cm, age ≥55 years, and positivity for Ex were the predictors of recurrence to the lateral nodes [2]. We abandoned routine pLND and the decision to perform pLND is now made by attending physicians on a case-by-case basis in consideration of the risk factors for lymph node recurrence. Generally, patients with two or more of the risk factors were regarded as candidates for pLND. In our previous study, we reported that pLND reduced recurrence to regional lymph nodes in a subset of patients with T ≥ 3 cm with positive Ex [10]. However, this study included patients treated during the period from 1997 to 2012, a time when ultrasound technology, including resolving power, significantly increased. Moreover, previous studies did not selectively evaluate recurrence to the lateral compartment dissected in the initial surgeries, and thus, it remains unclear whether pLND contributes to reducing recurrence to the lateral compartment. In the present study, we included patients who underwent surgery from 2007 to 2017 to investigate if pLND improved lateral compartment recurrence-free survival (RFS) and distant RFS.

## 2 Materials and methods

### 2.1 Patients

We conducted retrospective study that identified 3177 patients who were diagnosed with PTC (T ≥ 2 cm; cN0 or N1a, M0) on fine-needle aspiration cytology (FNAC) before surgery and underwent their initial surgery between 2007 and 2017 in our institution. All patients were negative for lateral node metastasis on preoperative imaging studies, primarily ultrasound examination. This retrospective study meeting the ethical standards of the World Medical Association Declaration of Helsinki was approved by the ethics committee of, which waived the need for informed consent. This trial has been registered on Research Registry (research registry 5466, https://www.researchregistry.com). The work has been reported in line with the STROCSS criteria [11].

### 2.2 Surgical designs

All patients underwent thyroidectomy, consisting of total thyroidectomy (2506 patients), subtotal thyroidectomy (18 patients), and lobectomy with isthmectomy (653 patients). All patients underwent at least therapeutic or prophylactic central node (level VI) dissection. The indications for pLND in the patients were made by attending physicians on a case-by-case basis in consideration of the risk factors for lymph node recurrence such as male, sex, T > 3 cm, age ≥55 years, and positive for Ex. All patients who underwent pLND were dissected at levels IIA, III, and IV. For a portion of patients, levels IIB and V were also dissected (partially or completely) according to the physicians’ discretion.

In this study, we defined significant extrathyroidal extension as an extrathyroidal extension corresponding to T3b and T4a based on AJCC TNM classification (8th edition) [12], which was evaluated on intraoperative findings. The “lateral compartment” in the pLND group was defined as the lateral compartment dissected in the initial surgery. In the non-pLND group, it was defined as the lateral compartment ipsilateral to the main primary lesions in the non-pLND group. Pathological lymph node metastasis was defined as lymph node metastasis based on postoperative pathology results.

### 2.3 Postoperative follow-up

Only 118 (3.7%) patients underwent radioactive iodine (RAI) ablation using RAI ≥30 mCi. All patients were followed by ultrasound at least once per year to check for lymph node recurrence. FNAC for suspicious nodes and thyroglobulin measurements in the needle washout used for FNAC were adopted to diagnose lymph node recurrence. Further, chest computed tomography scan and/or chest roentgenogram were performed for checking distant recurrence once a year under physicians’ discretion. The median follow-up period was 6.1 years (range, 0.1–12.0).

### 2.4 Statistical analysis

Variables were compared using the chi-squared test and the Fisher's exact test. The Kaplan-Meier method and log-rank test were used for analyses of time-dependent variables. The Cox regression model was also adopted for multivariate analysis. A p-value <0.05 was regarded as significant. We used StatFlex ver. 6.0 (Artec/Japan) software for these analyses.

## 3 Results

### 3.1 Clinical and pathological features in pLND and non-pLND groups

Table 1 summarizes the relationship between clinicopathological features according to whether or not pLND was performed (pLND and non-pLND groups). Ipsilateral or bilateral pLND was performed for 494 patients (15.5%). pLND was performed more frequently for patients with male sex, younger age (<55 years), T ≥ 3 cm, Ex positivity, and cN1a.

**Table 1:** Clinicopathological features of patients with and without prophylactic lateral neck dissection.

Characteristics		Prophylactic lateral neck dissection		p-value
		Yes	No	
Age	≥55 years	213 (43%)	1320 (49%)	0.01
	<55 years	281 (57%)	1363 (51%)	
Sex	Male	115 (23%)	416 (16%)	<0.01
	Female	379 (77%)	2267 (84%)	
cCentral(+)	Yes	157 (32%)	332 (12%)	<0.01
	No	337 (68%)	2351 (88%)	
pCentral(+)	Yes	349 (71%)	1559 (58%)	<0.01
	No	145 (29%)	1124 (42%)	
pLateral(+)	Yes	328 (66%)		
	No	166 (34%)		
Tumor size	≥3 cm	325 (66%)	371 (14%)	<0.01
	<3 cm	169 (34%)	2312 (86%)	
Ex	Yes	114 (23%)	215 (8%)	<0.01
	No	380 (77%)	2468 (92%)	
Total		494 (100%)	2683 (100%)	

cCentral(+): clinical central node metastasis. pCentral(+): pathological central node metastasis. pLateral(+): pathological lateral node metastasis. Ex: significant extrathyroidal extension.

### 3.2 Recurrent sites and events in pLND and non-pLND groups

Table 2 shows the organs where PTC recurred. Twenty-two and 61 patients showed recurrence to the central and lateral compartments, respectively. Twenty-six patients showed recurrence to other organs, including 14 who developed recurrence in the contralateral lateral lymph nodes, 4 who developed recurrence in the mediastinal lymph nodes, and 8 who developed recurrence in both areas subcutaneously. Subcutaneous recurrence was observed in 8 cases, 2 in the pLND group and 6 in the non-pLND group. The incidence showed no significant difference between the two groups. Simultaneous multiple recurrence was observed in 18 cases. Recurrence to distant organs such as the lung, bone, or brain was detected in 21, 2, and 1 patient, respectively. Five patients died of PTC.

**Table 2:** Recurrent sites in patients with and without prophylactic lateral neck dissection.

	Prophylactic lateral neck dissection		p-value
	Yes (n = 494)	No (n = 2683)	
Any site	23 (4.7%)	76 (2.8%)	0.03
Central LN^a^	8 (1.6%)	14 (0.5%)	0.01
Ipsilateral lateral LN^b^	11 (2.2%)	50 (1.9%)	0.59
Contralateral lateral LN^c^	4 (0.8%)	10 (0.4%)	0.25
Mediastinal LN^d^	2 (0.4%)	2 (0.1%)	0.12
Subcutaneous^e^	6 (1.2%)	2 (0.1%)	<0.01
Distant^f^	9 (1.8%)	15 (0.6%)	<0.01

Simultaneous multiple recurrence was observed in 18 cases.

a Central compartment lymph node recurrence.

b Ipsilateral lateral compartment lymph node recurrence.

c Contralateral lateral compartment lymph node recurrence.

d Mediastinal compartment lymph node recurrence.

e Subcutaneous recurrence.

f Distant recurrence.

### 3.3 Lateral compartment and distant RFS

**Fig. 1:**
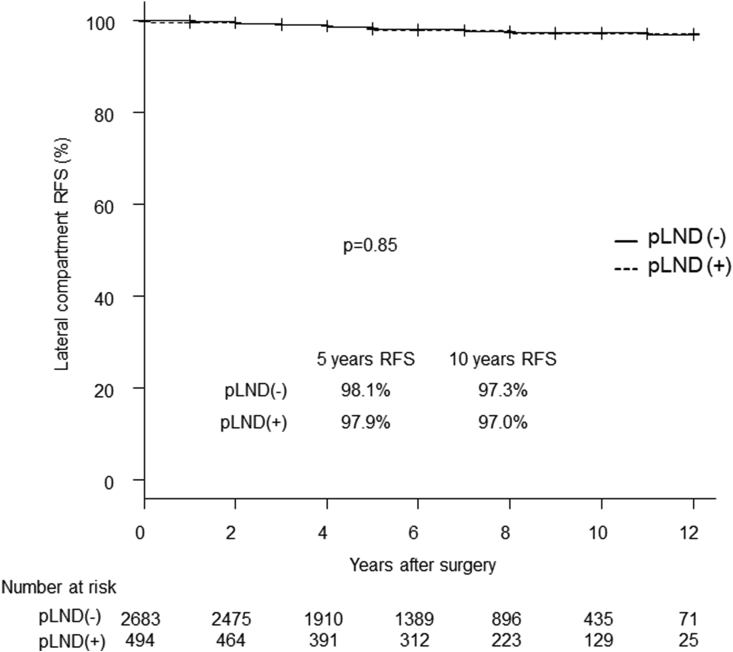
Kaplan-Meier curves for the lateral compartment RFS of the pLND and non-pLND groups. RFS: recurrence-free survival. pLND: prophylactic lateral neck dissection.

**Fig. 2:**
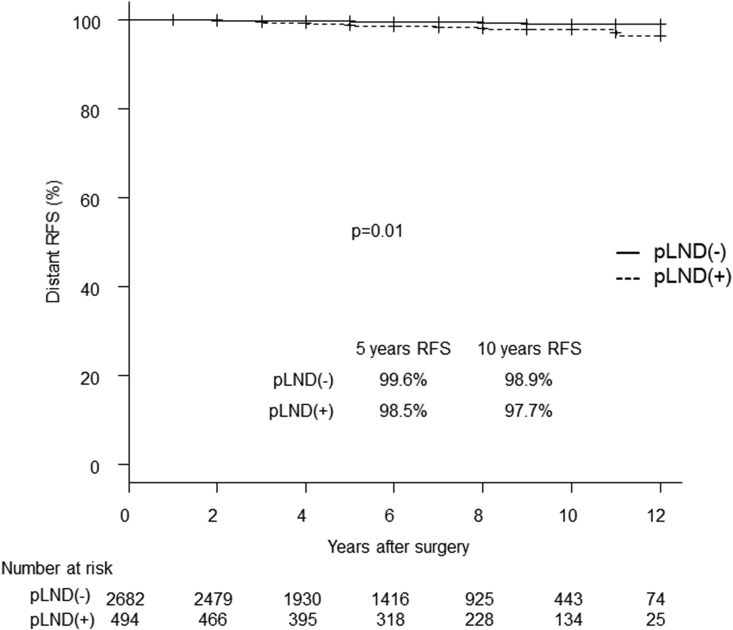
Kaplan-Meier curves for the distant RFS of the pLND and non-pLND groups. RFS: recurrence-free survival. pLND: prophylactic lateral neck dissection.

We then investigated the difference in recurrence to the lateral compartment between the pLND and non-pLND groups. In the analysis of the entire series, there was no significant difference in lateral compartment RFS between the 2 groups (Fig. 1). The non-pLND group had a significantly better prognosis regarding distant RFS than the pLND group (p = 0.01) (Fig. 2), which is consistent with less advanced disease seen in the former group (Table 1).

### 3.4 Multivariate analysis

On multivariate analysis, T ≥ 3 cm and positive Ex were independent prognostic factors for recurrence to the lateral compartment (Table 3). Further, we performed a subset analysis for patients in the pLND and non-pLND groups. In the pLND group, no significant risk factors could be identified, while in the non-pLND group, T ≥ 3 cm and positive Ex were independent predictors for lateral compartment recurrence (Table 4).

**Table 3:** Multivariate analyses of clinicopathological features for lateral compartment recurrence in the entire population (n = 3177).

	p-value	Hazard ratio(95% CI)	NOP (%)
Sex (male)	0.74	0.88(0.42–1.86)	531(16.7%)
Age (≥55 years)	0.27	1.34(0.80–2.23)	1533(48.3%)
Tumor ≥ 3 m	0.02	2.06(1.12–3.77)	696(78.1%)
Ex	<0.01	3.51(1.96–6.30)	329(10.4%)
pLND performed	0.13	0.56(0.27–1.19)	494(15.5%)

CI: confidence interval. pLND: prophylactic lateral neck dissection. NOP: number of patients. Ex: significant extrathyroidal extension.

**Table 4:** Multivariate analyses of clinicopathological features for lateral compartment recurrence in patients with and without prophylactic lateral neck dissection.

	pLND group		NOP (%)	Non-pLND group		NOP (%)
	p-value	Hazard ratio(95% CI)		p-value	Hazard ratio(95% CI)	
Sex (male)	0.37	0.39(0.05–3.08)	115(23.3%)	0.99	1.00(0.45–2.24)	416(15.5%)
Age (≥55 years)	0.17	2.42(0.68–8.60)	213(43.1%)	0.44	1.26(0.71–2.23)	1320(49.2%)
Tumor ≥ 3 cm	0.20	0.45(0.14–.51)	325(65.8%)	<0.01	2.95(1.60–5.44)	371(13.8%)
Ex	0.40	1.72(0.50–6.32)	114(23.1%)	<0.01	4.10(2.17–7.76)	215(8.0%)

CI: confidence interval. pLND: prophylactic lateral neck dissection. NOP: number of patients. Ex: significant extrathyroidal extension.

### 3.5 Subset analysis

**Fig. 3:**
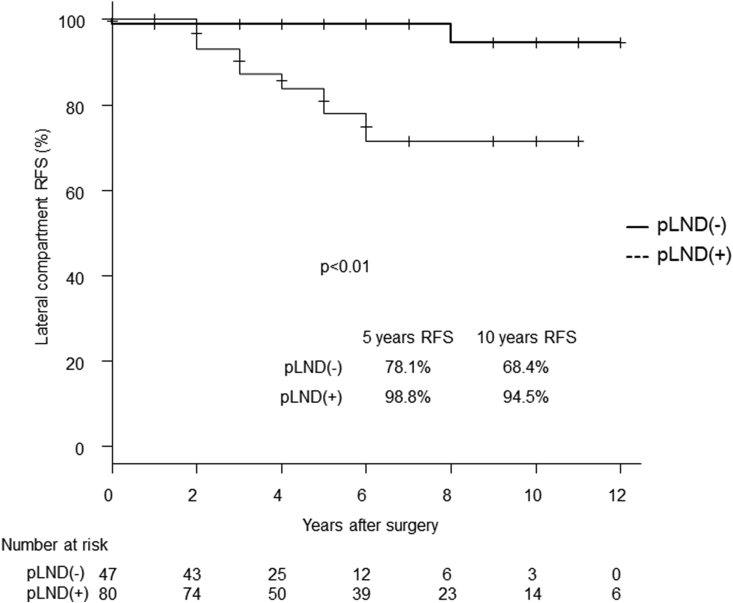
Kaplan-Meier curves for the lateral compartment RFS of the pLND and non-pLND groups with a tumor size ≥3 cm with positive extrathyroidal extension.

**Fig. 4:**
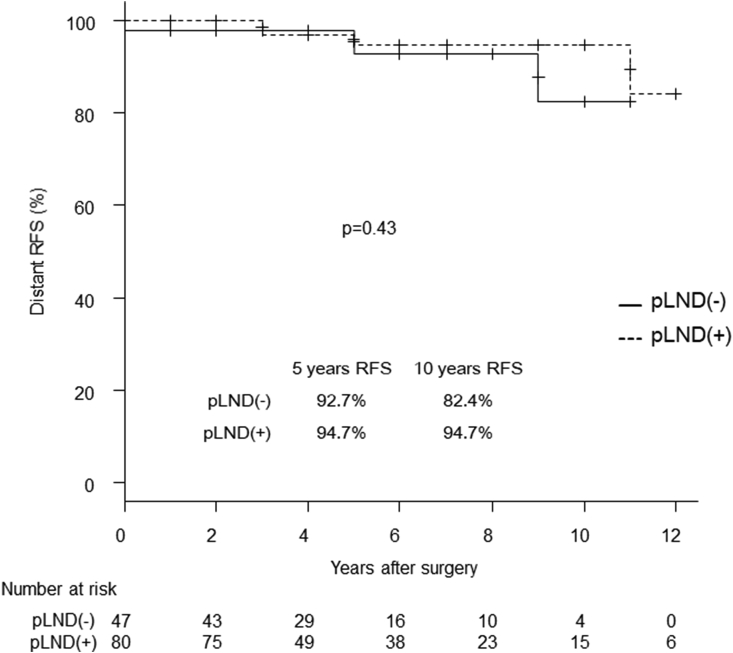
Kaplan-Meier curves for distant RFS of the pLND and non-pLND groups with a tumor size ≥3 cm with positive extrathyroidal extension.

In the subset of T ≥ 3 cm with positive Ex (n = 127), the lateral compartment RFS rate of the pLND group was significantly better than that of the non-pLND group (p < 0.01) (Fig. 3). In this subset, performing pLND reduced the lateral compartment recurrence rate by 20.7% during the 5-year follow-up (Fig. 3). However, distant RFS did not differ between the 2 groups (Fig. 4).

## 4 Discussion

In this study, we demonstrated that 1) pLND significantly improved lateral node RFS for PTC ≥3 cm with positive Ex by 20.7% during the 5-year follow-up, and 2) pLND did not improve distant RFS of PTC patients.

pLND requires an extensive skin incision, is time-consuming, and shows significant adverse events for patients such as pain and discomfort of the neck, although postoperative stretching exercises can reduce patients’ symptoms regarding neck discomfort [13]. Moreover, therapeutic LND may be complicated by adverse events such as chyle leakage, Horner syndrome, and injury of the jugular vein, vagal nerve, phrenic nerve, and accessory nerve; however, these events are rare in the prophylactic setting. Our institution is a hospital specializing in the thyroid, and all surgeons are experts in thyroid surgery. In our series, chyle leakage, phrenic nerve paralysis, and Horner syndrome occurred in 0.4%, 0.2%, and 0.2%, respectively, of patients in the pLND group. If pLND were performed by non-experts, the incidence of adverse events would be higher. Therefore, pLND should be performed only in patients for whom this surgical procedure is considered beneficial, and routine pLND is not appropriate.

Although lateral node-negative for PTC, approximately 40% of patients with a tumor diameter ≤1 cm had lateral lymph node metastasis on postoperative pathology, the lymph node recurrence rate is 0% in 10 years [2]. We found that routine pLND did not improve the outcome, and that male sex, T ≥ 3 cm, age ≥55 years, and positivity for Ex were the predictors of recurrence to the lateral nodes [2]. In our previous study, we reported that pLND reduced recurrence to regional lymph nodes in a subset of T ≥ 3 cm with positive Ex [10]. However, it was unclear if these two factors were predictors of recurrence to “ipsilateral” lateral lymph node to the dissected side. In this study, we investigated PTC cases suitable for pLND, and showed that pLND for T ≥ 3 cm with positive Ex could significantly improve the “ipsilateral” lateral compartment RFS. Surprisingly, in this subset, performing pLND reduced the lateral compartment recurrence rate by as much as 20.7% during the 5-year follow-up. Although lymph node recurrence is not always life threatening, it causes anxiety for both patients and physicians. Despite the adverse events described above, we can conclude that pLND could be recommended for patients with T ≥ 3 cm with positive Ex to reduce lateral node recurrence. Our multivariate analysis demonstrated that in the pLND group, no predictor of lateral node recurrence could be identified; however, in the non-pLND group, T ≥ 3 cm and positive Ex were regarded as independent risk factors of lateral node recurrence. These results strongly support the value of using pLND in selected patients with these risk factors.

In contrast, pLND did not reduce distant recurrence. In our series, the number of patients who showed distant recurrence was small at 24 (0.8%). Since only 5 patients (0.2%) died of PTC, we were not able to analyze the effect of pLND on disease specific survival. However, since pLND did not improve distant RFS, the procedure unlikely improves disease specific survival.

This study has some limitations. First, this was a retrospective study, and patients were not randomized into the pLND and non-pLND groups; therefore, patient characteristics were significantly different between the pLND and non-pLND groups. Second, although our series included a considerably large number of patients with high-risk features, the number of patients who underwent adjuvant RAI therapy or RAI ablation using RAI ≥30 mCi was small at 118 (3.7%). However, in this series, regional lymph node recurrence was 2.5% (79/3177) overall. In the previous series, only 0.2% of patients (21/10366) underwent adjuvant RAI therapy or RAI ablation using RAI ≥ 30 mCi and regional lymph node recurrence was only 2.6% (273/10366) [10]. These results may suggest that RAI does not much contribute to the prevention of regional lymph node recurrence.

## 5 Conclusions

In summary, although pLND is not beneficial for all PTC patients, it significantly improves lateral node RFS for patients with T ≥ 3 cm with positive Ex. For such patients, pLND at initial surgery may be considered after obtaining informed consent from patients to reduce the recurrence to the lateral compartment and avoid second surgery.

## Declaration of competing interestCOI

No competing financial interests exist.

## Source of funding

No funding exists for present study.

## Ethnical approval

The Ethical Committee at Kuma Hospital approved the present retrospective study of the clinical outcomes. The institutional review board at Kuma Hospital waived obtaining informed consent from the patients due to the retrospective nature of this study.

## Author contributions

Study design: Akira Miyauchi, Yasuhiro Ito.

Data collections: Makoto Fujishima, Minoru Kihara, Akihiro Miya.

Data analysis: Makoto Fujishima, Takumi Kudo.

Writing: Makoto Fujishima.

Revision and final manuscript approval: Akira Miyauchi.

## Provenance and peer review

Not commissioned, externally peer reviewed.
